# Association of American Medical Editors

**Published:** 1886-03

**Authors:** 


### ASSOCIATION OF AMERICAN MEDICAL EDITORS.

THIS Society meets annually at the time and place of meeting of the A. M. A. The convention will be held this year at St. Louis, on the Monday evening preceding the meeting of the American Medical Association, which takes place *the first Tuesday* in May, being the fourth day, and *not* on the *second* Tuesday, as so persistently stated by most of our exchanges. Our genial contemporary of the *American Lancet* proposes that the event be commemorated by a dinner, at which the business of the meeting can be discussed along with the viands; and our sage friend of the amalgamated *American Practitioner and News* goes him better by adding that the papers can be listened to by the members while they enjoy their *post prandial* Havanas. We wish to second the motion and the amendment, and now call on our confreres of the press to vote for both. Let the arrangement committee, of which Dr. Atwood is chairman, make all preparations for our dinner; and let it be understood that each guest is to pay for his own dinner. In this way the genial editors can combine business with pleasure, and have a good time generally, and nobody will feel the cost. The committee can arrange the programme, which should be announced prior to assembling at St. Louis. We sincerely hope to see a fuller attendance than is usual on such occasions.

